# Androgen receptor signalling impairs docetaxel efficacy in castration-resistant prostate cancer

**DOI:** 10.1038/s41416-020-01105-y

**Published:** 2020-09-29

**Authors:** Lisanne Mout, Jan M. Moll, Mingqing Chen, Eleonora S. de Morrée, Corrina M. A. de Ridder, Alice Gibson, Debra Stuurman, Ashraf Aghai, Sigrun Erkens-Schulze, Ron H. J. Mathijssen, Alex Sparreboom, Ronald de Wit, Martijn P. Lolkema, Wytske M. van Weerden

**Affiliations:** 1 Department of Medical Oncology Erasmus MC-Cancer Institute, Dr. Molewaterplein 40, 3015 GD Rotterdam, The Netherlands; 2 Department of Urology Erasmus University MC, Dr. Molewaterplein 40, 3015 GD Rotterdam, The Netherlands; 3 Division of Pharmaceutics College of Pharmacy, The Ohio State University, 217 Lloyd M. Parks Hall, 500 West 12th Avenue, Columbus, OH 43210 USA

**Keywords:** Prostate cancer, Chemotherapy

## Abstract

Androgen receptor (AR) signalling drives neoplastic growth and therapy resistance in prostate cancer. Recent clinical data show that docetaxel combined with androgen deprivation therapy improves outcome in hormone-sensitive disease. We studied whether testosterone and AR signalling interferes with docetaxel treatment efficacy in castration-resistant prostate cancer (CRPC). We found that testosterone supplementation significantly impaired docetaxel tumour accumulation in a CRPC model, resulting in decreased tubulin stabilisation and antitumour activity. Furthermore, testosterone competed with docetaxel for uptake by the drug transporter OATP1B3. Irrespective of docetaxel-induced tubulin stabilisation, AR signalling by testosterone counteracted docetaxel efficacy. AR-pathway activation could also reverse long-term tumour regression by docetaxel treatment in vivo. These results indicate that to optimise docetaxel efficacy, androgen levels and AR signalling need to be suppressed. This study lends evidence for continued maximum suppression of AR signalling by combining targeted therapeutics with docetaxel in CRPC.

## Background

The treatment of advanced or metastatic prostate cancer (PCa) is focussed around androgen deprivation therapy (ADT), as testosterone and dihydrotestosterone promote neoplastic behaviour of PCa cells through androgen receptor (AR) signalling. While ADT almost invariably induces disease regression, the majority of prostate tumours will at some point become resistant due to AR-pathway aberrations.^1–3^ For patients who progress while receiving ADT, also referred to castration-resistant prostate cancer (CRPC), docetaxel is the standard first-line chemotherapy. Recently, the combination of ADT with docetaxel has also been introduced in the metastatic castrate-naive setting as this combination significantly increased overall survival.^4,5^ Conversely, docetaxel without ADT after radical prostatectomy did not delay disease recurrence.^6^ These clinical trials suggest that the antitumour efficacy of docetaxel in castrate-naive PCa (CNPC) is improved by inhibition of AR-pathway signalling. In this study, we explore the impact of sustained androgen levels and/or AR-pathway signalling on docetaxel efficacy in CRPC. Moreover, we studied the underlying mechanisms of AR-pathway activation on docetaxel treatment efficacy by examining docetaxel tumour accumulation, target engagement and cell death induction.

## Methods

The AR-positive CRPC patient-derived cell line models, PC346C-DCC-K and VCaP-DCC-E, were derived from the hormone-sensitive prostate cancer cell lines, PC346C and VCaP, respectively, through long-term propagation in castrate conditions.^7^ Both PC346C-DCC-K and the parental cell line PC346C do not express AR variants (Supplementary Fig. 1),^8^ while VCaP-DCC-E shows increased expression of AR-V7 compared to VCaP. Furthermore, these cell lines do not express ABCB1 (P-glycoprotein), which has been shown to induce multidrug resistance.^9^ For in vivo experiments, NMRI nu/nu male mice were subcutaneously inoculated with PC346C-DCC-K cells and surgically castrated once tumours established (Supplementary Methods). After 1 week, mice received a testosterone pellet or treatment control. One injection of docetaxel (33 mg/kg) or NaCl was given the following day. Tumour volume was monitored weekly by callipers and mice were euthanised before the humane endpoint was reached by cervical dislocation (details in Supplementary Methods).^10^ For in vivo accumulation studies, tumours were obtained 3 days after docetaxel treatment. Tumour samples were used to determine docetaxel accumulation by liquid chromatography with tandem mass spectrometry,^11,12^ α-tubulin acetylation by western blot and cell death by terminal deoxynucleotidyl transferase dUTP nick-end labeling (TUNEL) staining. Uptake of docetaxel by OATP1B3 was studied in Hek293T cells transiently expressing *SLCO1B3*, uptake of C^14^-docetaxel was measured in the presence or absence of testosterone. To assess the impact of AR-pathway stimulation on docetaxel sensitivity, cell viability assays were conducted. We exposed PC346C-DCC-K and VCaP-DCC-E cells in vitro to a dose range of docetaxel with or without androgens.

## Results

**Fig. 1:**
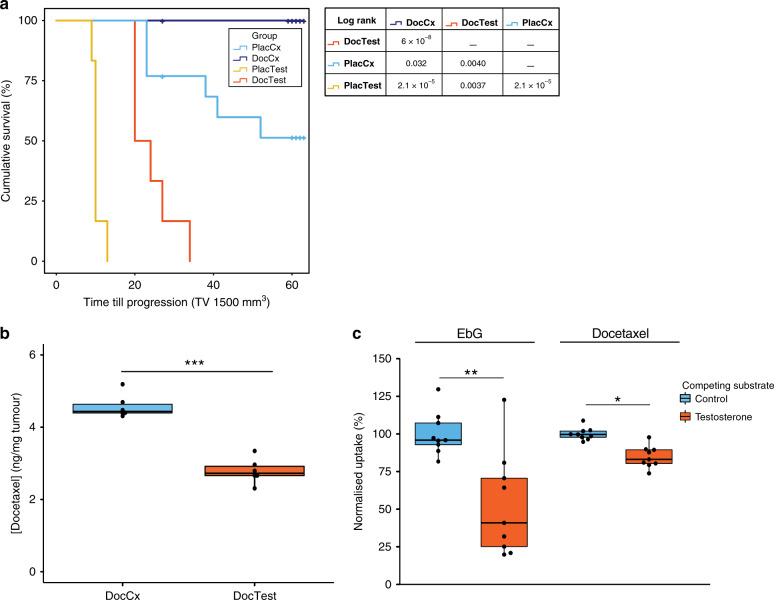
Testosterone supplementation strongly impairs docetaxel efficacy and accumulation. **a** Impact of testosterone supplementation on docetaxel treatment in tumour bearing mice (PC346C-DCC-K). Kaplan–Meier curve depicting the cumulative survival in the four treatment groups; Docetaxel (once 33 mg/kg i.v.) or placebo in castrate (DocCx *n* = 13 and PlacCx *n* = 13, respectively) or testosterone-supplemented mice (PlacTest *n* = 6 and DocTest *n* = 6, respectively). Time till progression was calculated from the day of docetaxel treatment until tumours exceeded a volume of 1500 mm^3^. Mice were censored when tumours did not reach 1500 mm^3^ in size during the maximum follow-up of 60 days. One mouse in the DocCx treatment group was euthanised due to continued weight loss after treatment. Results from the pair-wise comparison using the log-rank test are shown in the table. **b** Docetaxel accumulation in PC346C-DCC-K tumours obtained 3 days after treatment in castrate or testosterone-supplemented mice (DocCx *n* = 6 and DocTest *n* = 6, respectively). ***P < 0.0001. **c** Normalised uptake of ^3^H-estradiol-17β-d-glucuronide (EbG) and C^14^-docetaxel in Hek293T cells transiently expressing *SLCO1B3*, pre/coincubated with 200 µM testosterone or vehicle control. Uptake of EbG and docetaxel was normalised to uptake in *SLCO1B3*-expressing cells in the absence of testosterone, and EbG was used as a positive control for OATP1B3-mediated uptake. Shown are the individual normalised uptake values (*n* = 9) obtained from three experiments. **P < 0.01 and *P < 0.05, respectively.

**Fig. 2:**
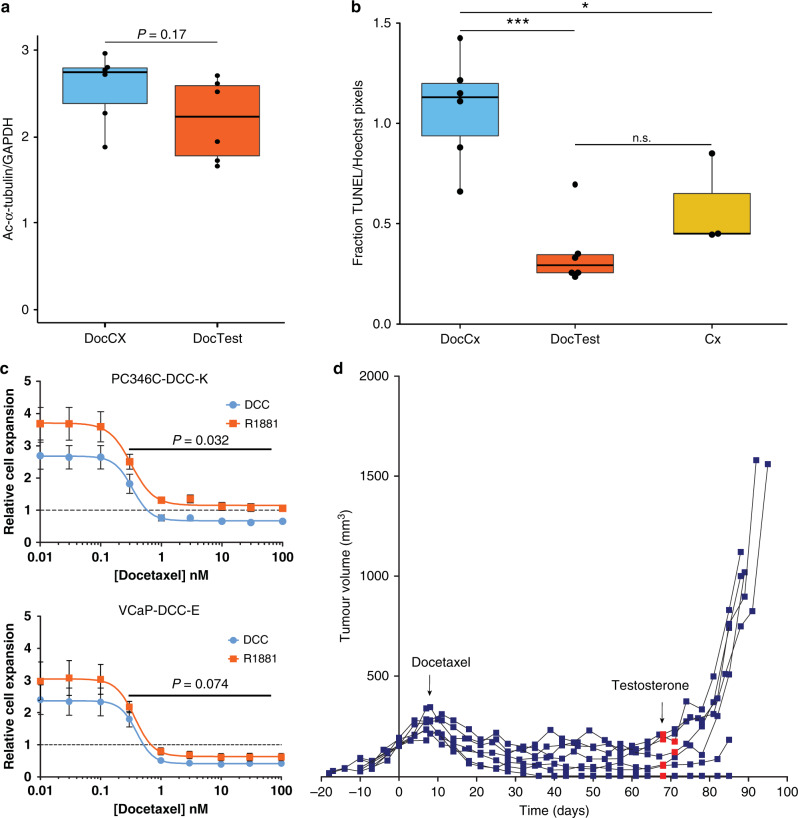
Testosterone and AR-pathway activity interfere with docetaxel-induced target engagement and cell death. **a** Acetylated-α-tubulin as a measurement for tubulin stabilisation in PC346C-DCC-K tumours obtained 3 days after docetaxel treatment, in castrate and testosterone-supplemented mice (DocCx *n* = 6 and DocTest *n* = 6, respectively). Acetylated-α-tubulin signal intensity was obtained by immunoblotting individual tumour samples and normalised to GAPDH loading control. Statistical comparison was performed by a two-sided *T* test. **b** Quantification of TUNEL stainings in short-term docetaxel-treated PC346C-DCC-K tumours from castrate and testosterone-supplemented mice (DocCx *n* = 6 and DocTest *n* = 6, respectively). TUNEL signal was compared to three tumours obtained from castrate mice (Cx) with no/low docetaxel accumulation (≤0.1 ng/mg tumour) after short-term treatment. Fraction of TUNEL-positive pixels was normalised to Hoechst signal, and data plotted is the median TUNEL-positive pixels of the individual tumour samples. ***P < 0.001 and *P < 0.05. n.s. Not significant. **c** Impact of androgen supplementation (R1881; orange data points) on docetaxel response as compared to androgen-deprived culture conditions (DCC; blue data points). Docetaxel sensitivity was assessed in the AR-positive CRPC cell lines PC346C-DCC-K (top panel) and VCaP-DCC-E (lower panel) (both *n* = 3). Docetaxel response was normalised to cell density at the start of docetaxel treatment (dashed line) and plotted as relative cell expansion. Data were fitted using a non-linear curve fit to compare the two culture conditions; *P* values are displayed. **d** Individual tumour growth curves of docetaxel-treated PC346C-DCC-K tumours under castrate conditions (from day 0, *n* = 7). Sixty days after docetaxel treatment, mice were supplemented with a testosterone implant (red data points) and tumour growth was monitored until tumours exceeded 1000 mm^3^, or a maximum follow-up of 21 days.

We first investigated the effect of androgen signalling on the antitumour efficacy of docetaxel in the AR-positive CRPC model PC346C-DCC-K (Supplementary Fig. 1). In all castrate mice, docetaxel treatment resulted in long-term and complete tumour regression (Fig. 1a). While in testosterone-supplemented mice, docetaxel treatment only induced a short-term tumour response, after which all tumours rapidly progressed. The partial response to docetaxel in these mice shows that testosterone interfered with docetaxel treatment efficacy. We therefore examined docetaxel tumour accumulation in the presence of testosterone, as we previously identified that impaired uptake of taxane chemotherapeutics correlates with treatment resistance.^11^ Indeed, testosterone-supplemented mice displayed a 40% reduction of docetaxel tumour levels: median of 2.8 ng/mg tumour from testosterone-supplemented mice versus 4.4 ng/mg in androgen-deprived animals (*P* value < 0.001; Fig. 1b). Moreover, testosterone supplementation resulted in strong AR-pathway activation in PC346C-DCC-K tumours as exemplified by increased prostate-specific antigen production, AR nuclear localisation, AR target gene expression and tumour cell proliferation (Supplementary Fig. 2). To study the underlying mechanism of impaired docetaxel tumour accumulation, we examined the impact of testosterone on docetaxel uptake by the influx drug transporter OATP1B3 (encoded by *SLCO1B3*). *SLCO1B3* is frequently overexpressed in prostate cancer, and docetaxel and testosterone are both OATP1B3 substrates^13–16^ (Supplementary Fig. 3). We found that in Hek293T cells expressing *SLCO1B3*, pre/coincubation with testosterone, decreased docetaxel uptake by 17% (*P* value = 0.004; Fig. 1c). This implicates that testosterone competes with docetaxel for OATP1B3-mediated uptake, resulting in reduced docetaxel tumour accumulation. As testosterone supplementation interferes with docetaxel accumulation, this will likely affect tubulin target engagement and subsequent stabilisation. We therefore investigated acetylation of α-tubulin, which is a result of taxane-mediated tubulin stabilisation.^17^ Indeed, α-tubulin acetylation was significantly correlated with docetaxel tumour accumulation levels (Supplementary Fig. 4a, b). Moreover, we observed a trend towards decreased tubulin stabilisation in PC346C-DCC-K tumours from short-term docetaxel-treated mice supplemented with testosterone (19% decrease, *P* = 0.17; Fig. 2a and Supplementary Fig. 4c). Overall, these data confirm that testosterone reduces docetaxel target engagement as a result of impaired tumour accumulation. To further assess docetaxel activity, we quantified docetaxel-induced cell death by TUNEL. Testosterone interfered with docetaxel-induced cell death, as shown by the strong reduction in TUNEL staining compared to docetaxel-treated castrate mice (*P* ≤ 0.001; Fig. 2b and Supplementary Fig. 5). Moreover, TUNEL staining in tumours from docetaxel-treated mice supplemented with testosterone was not significantly different to untreated controls (*P* value = 0.36). Therefore, tubulin stabilisation by docetaxel treatment in testosterone-supplemented mice, albeit reduced, did not translate into cell death induction. We thus hypothesised that AR-pathway activation by testosterone further abrogates docetaxel efficacy. Indeed, a small but consistent survival advantage was achieved by R1881 under effective docetaxel concentrations in AR-positive CRPC cell lines (Fig. 2c; > 0.3 nM docetaxel with *P* = 0.032 and 0.073 for PC36C-DCC-K and VCaP-DCC-E, respectively). R1881 did not impact docetaxel response in AR-negative prostate cancer (PCa) cells (Supplementary Fig. 6). This indicates that androgen stimulation can protect AR-positive CRPC cells from docetaxel-induced cell death. The increased cell viability under docetaxel treatment led us to investigate whether AR-pathway stimulation alone could re-induce proliferation in vivo. We therefore repeated the docetaxel treatment in castrate mice as depicted in Fig. 1a and investigated the impact of AR-pathway activation. Testosterone-induced strong AR-pathway stimulation, as shown by AR nuclear localisation and target gene expression, which led to rapid outgrowth in five out of seven PC346C-DCC-K tumours (Fig. 2d and Supplementary Fig. 7). Overall, AR-pathway activation was able to overcome long-term tumour regression induced by docetaxel treatment.

## Discussion

In this study, we investigated the impact of testosterone and AR-pathway stimulation on the antitumour efficacy of docetaxel in CRPC xenografts and cell lines. Testosterone impaired docetaxel antitumour activity through two different modes of action: interference with docetaxel tumour accumulation, thereby reducing tubulin stabilisation and AR-pathway activation protecting cells from docetaxel-induced cell death (Fig. 1b). Reduced levels of docetaxel tumour accumulating by testosterone are, at least partially, mediated through competition for OATP1B3 uptake (Fig. 1c). OATP1B3 is an influx transporter of particular interest, because it is frequently overexpressed in CNPC and metastatic CRPC (mCRPC) compared to normal adjacent prostate.^18–20^ Moreover, OATP1B3 seems to have a dual character in PCa, while *SLCO1B3* overexpression has been linked to testosterone uptake; downregulation was correlated with docetaxel resistance in vivo.^13,21^ We previously showed that cabazitaxel efficacy and tumour accumulation are also impaired by testosterone, indicating that testosterone impacts taxane efficacy in general.^8^

The current study explored the impact of reduced taxane tumour accumulation on tubulin target engagement. The absence of acetylated α-tubulin has previously been described as a biomarker of paclitaxel resistance and was restored upon re-sensitisation.^17^ Here, we found that docetaxel accumulation strongly correlated with acetylated α-tubulin signal in short-term docetaxel-treated tumours. Moreover, tubulin acetylation tended to be decreased in mice receiving docetaxel together with testosterone (Fig. 2a). Although CRPC xenografts from testosterone-supplemented docetaxel-treated mice still displayed substantial tubulin stabilisation, this did not translate into increased cell death induction (Fig. 2b). These results led us to conclude that testosterone activation of the AR pathway compromised the antitumour activity of docetaxel. This was in part demonstrated by the impact of R1881 supplementation on docetaxel sensitivity in vitro in two AR-expressing CRPC cell lines. Of note, the VCaP-DCC-E cell line is positive for AR-V7, while PC346C-DCC-K does not express AR variants (Supplementary Fig. 1),^8^ this implicates that regardless of variant expression, AR-pathway activity interferes with taxane efficacy. In addition, AR-pathway activation by testosterone stimulated regrowth of docetaxel-treated dormant tumours (Fig. 2d). Recent clinical findings, albeit retrospective analyses, also suggest an interaction between taxane efficacy and testosterone. These studies showed that CRPC patients with suboptimal testosterone serum levels had significantly shorter progression-free survival following docetaxel treatment.^22,23^

Our data strongly suggest that taxane efficacy can be optimised by maintaining suppression of testosterone levels in order to minimise interference with docetaxel tumour accumulation and block AR-driven prosurvival signalling. Several phase II/III trials are currently combining (novel) AR-targeted therapies with taxanes in mCRPC.^24^ Our findings lend support to the potential benefit of combining docetaxel with additional blocking of AR signalling in mCRPC.

## Supplementary information

Supplementary files

## Data Availability

The PCa cell lines PC346C-DCC-K and VCaP-DCC-E and PC339 will be made available for academic institutions under the Erasmus MC Biological Uniform Material Transfer Agreement.
