# Supplementary material for: Androgen receptor signalling impairs docetaxel efficacy in castration-resistant prostate cancer
Source: Br J Cancer. 2020 Sep 29;123(12):1715–9. doi: 10.1038/s41416-020-01105-y (PMC7722857; doi:10.1038/s41416-020-01105-y)
Supplement: Supplementary file 1 — Supplementary files [file 41416_2020_1105_MOESM1_ESM.pdf]

Supplementary figure 1

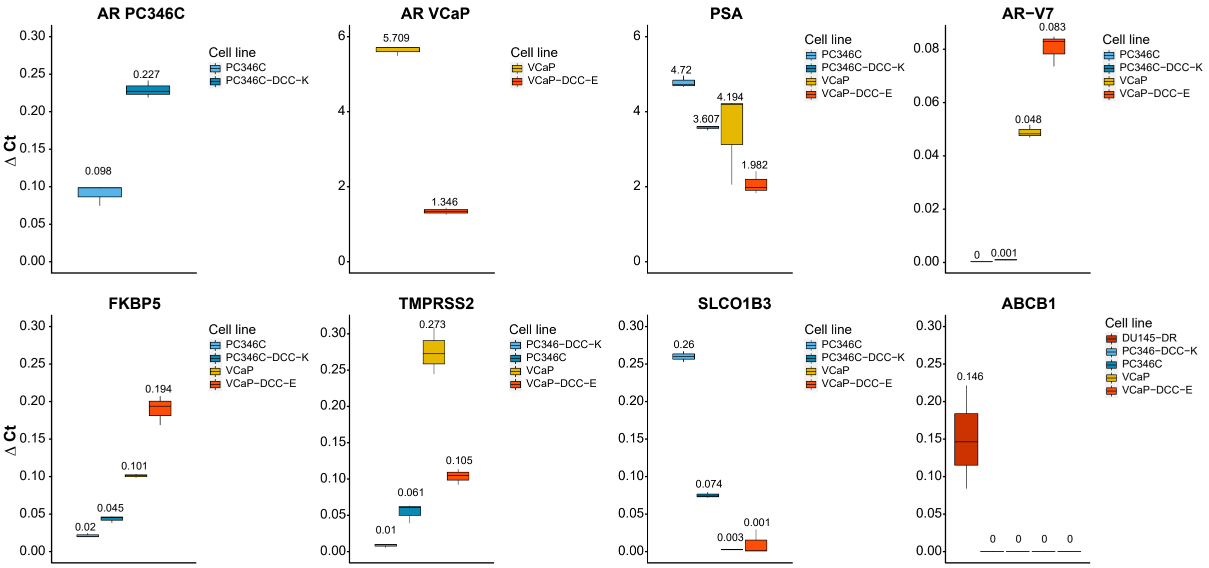

Supplementary figure 2

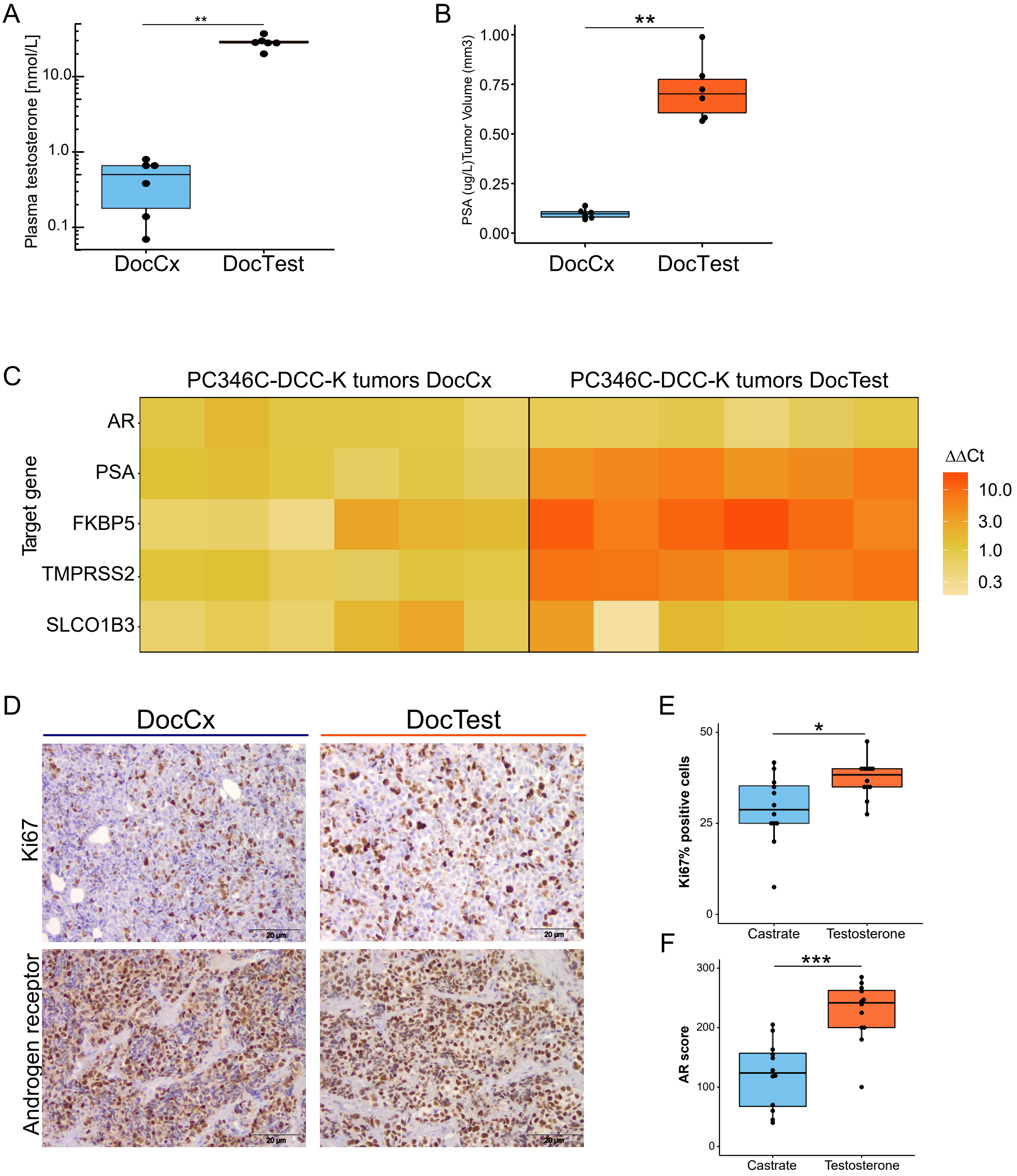

Supplementary figure 3

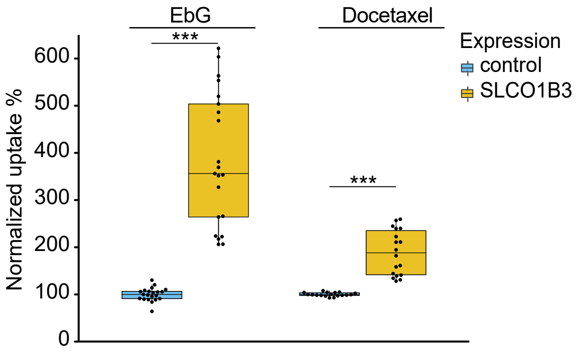

Supplementary figure 4

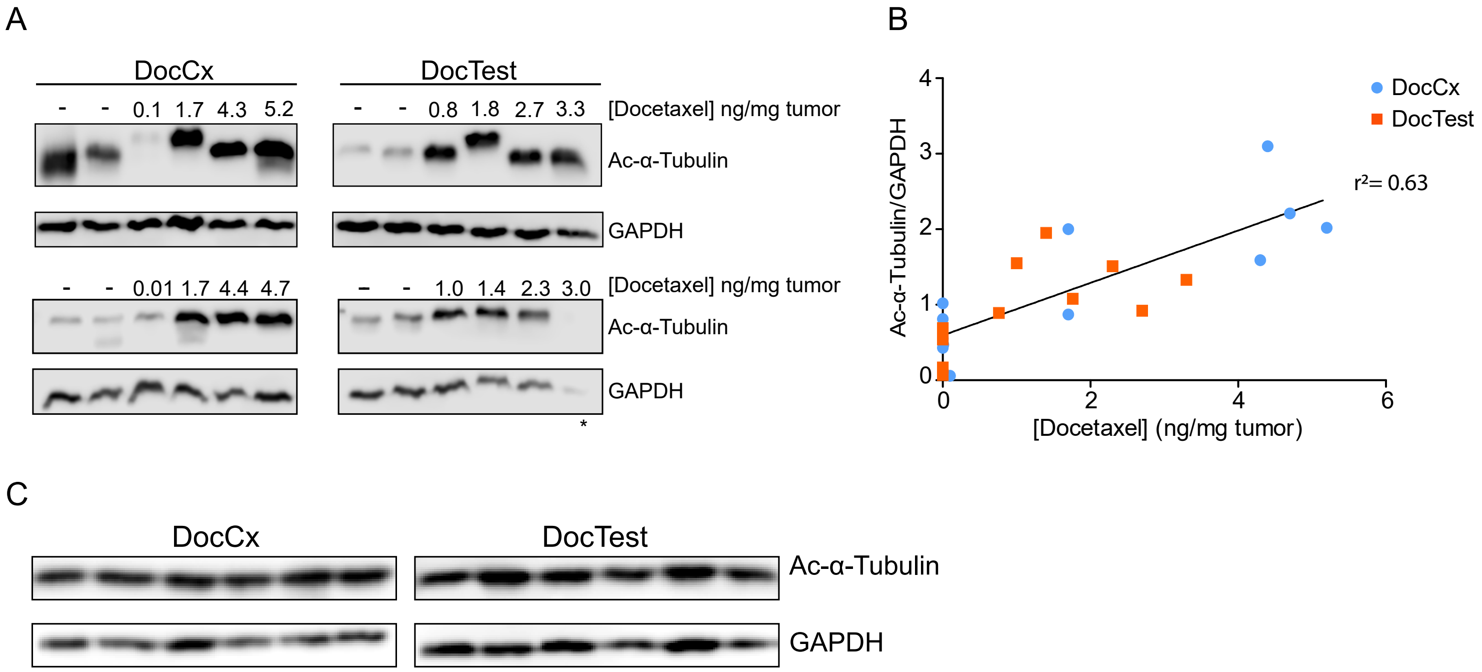

Supplementary figure 5

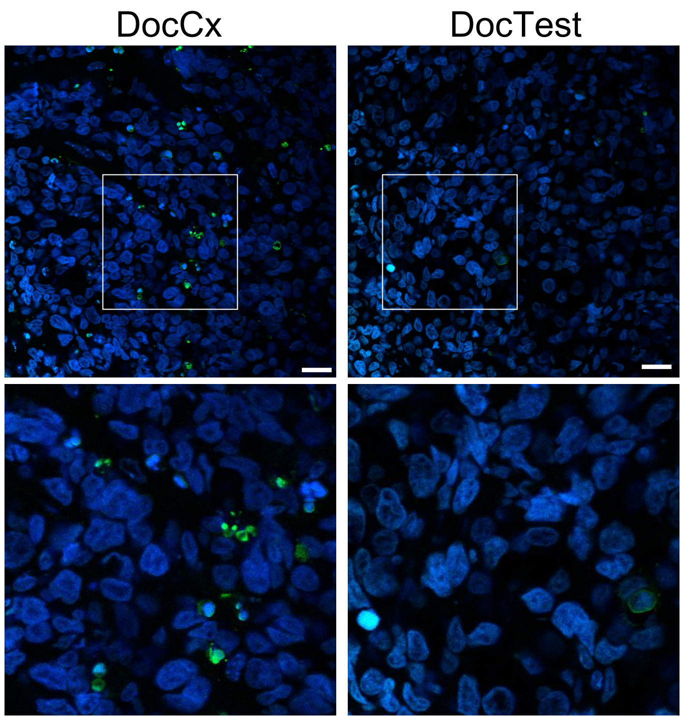

Supplementary figure 6

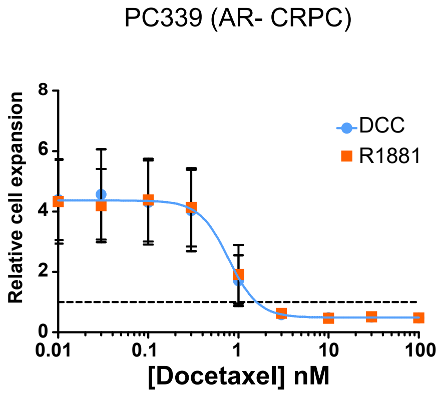

Supplementary figure 7

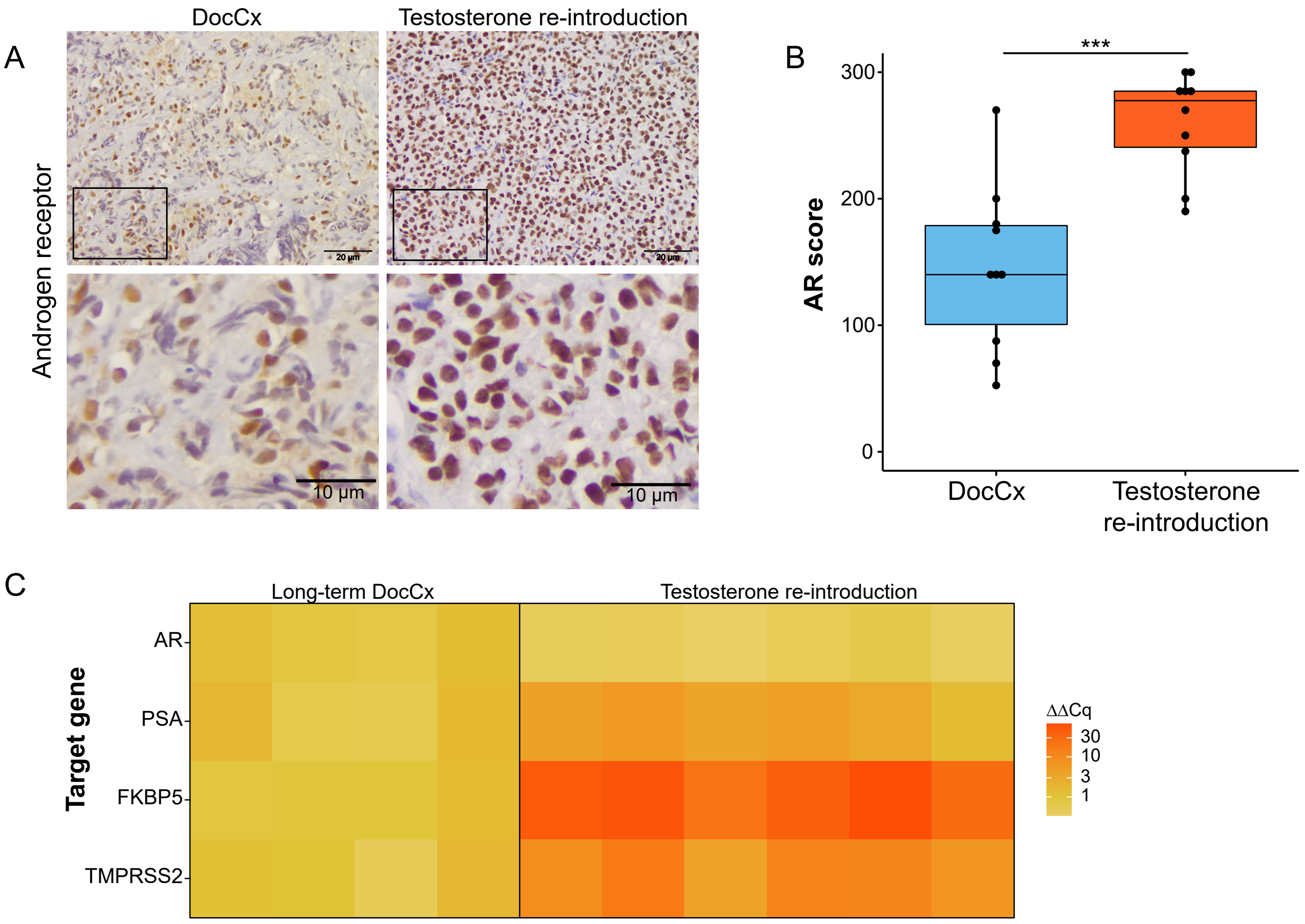

Supplementary figure 1: Expression of relevant genes in prostate cancer cell line models used. Gene expression of the *AR*, *AR*-target genes and *SLCO1B3* in PC346C-DCC-K, VCaP-DCC-E and the corresponding parental cell lines as assessed by qRT-PCR. Additionally the expression of the drug transporter ABCB1 was assessed, for which a positive control (DU145-DR) was included. The hinges of the boxplots represent the 25th and 75th percentile with the median and the whiskers represent 1.5x the interquartile range (IQR). For each gene three technical replicates were included and expression was normalized to two household genes using the $2^{-\Delta Ct}$ method.

Supplementary figure 2: Testosterone supplementation in mice leads to increased plasma testosterone, prostate specific antigen levels, AR target gene expression and cancer cell proliferation. A) Testosterone plasma levels in short-term docetaxel treated castrate and testosterone supplemented tumour bearing mice (DocCx n=6 and DocTest n=6 resp.). Plasma samples were collected four days after mice received silastics implants containing testosterone or empty controls. The hinges of the boxplots represent the 25th and 75th percentile with the median and the whiskers represent 1.5x the interquartile range (IQR), ** indicates $P<0.01$. **B)** Normalized PSA plasma levels in short-term docetaxel treated castrate and testosterone supplemented tumour bearing mice (DocCx n=6 and DocTest n=6 resp.). Plasma samples were collected four days after mice received silastics implants containing testosterone or empty controls. PSA values were normalized to tumour volumes. The hinges of the boxplots represent the 25th and 75th percentile with the median and the whiskers represent 1.5x the IQR, ** indicates $P<0.01$. **C)** Gene expression of the *AR* and *AR*-target genes expressed in PC346C-DCC-K tumours after short-term docetaxel treatment in castrate and testosterone supplemented mice (both n=6). Heatmap depicts gene expression as assessed by qRT-PCR of the individual genes, with each row being one tumour sample. Plotted is the geomean of three technical replicates, normalized to two household genes and gene expression in tumours from castrate mice, using the $2^{-\Delta\Delta Ct}$ method. **D)** Representative immunohistochemistry images of PC346C-DCC-K tumours, obtained three days after docetaxel treatment in castrate or testosterone supplemented mice (DocCx and DocTest resp.). Tumour sections were stained for the AR and cell cycle marker Ki67, scale-bars represent 20 µm and 10 µm for the enlarged images. Images were scored by two independent viewers for Ki67 **(E)** percentage positive cells and AR **(F)** percentage positive cells which was multiplied by the AR staining intensity (scale 0-3).

Supplementary figure 3: Docetaxel is a substrate for OATP1B3 mediated uptake. Uptake of C^{14}-Docetaxel and ^{3}H-estradiol-17β-D-glucuronide (^{3}H-EbG) in Hek293T cells transiently expressing *SLCO1B3* and normalized to cells expressing the vector backbone. ^{3}H-EbG uptake was used as a positive control for OATP1B3 uptake. Shown are the individual normalized uptake levels (Docetaxel n=18 and EbG n=21) obtained from six or seven individual experiments. The hinges of the boxplots represent the 25th and 75th percentile with the median and the whiskers represent 1.5x the interquartile range (IQR), *** indicates $P<0.001$.

Supplementary figure 4: Docetaxel tumour accumulation levels correlate with α-tubulin acetylation. A) Immunoblots stained for acetylated α-tubulin in short-term docetaxel treated tumours. Shown are six biological replicates of PC346C-DCC-K tumours with varying levels of docetaxel tumour accumulation.

Individual docetaxel concentrations are indicated above each lane. Lane marked by * was excluded from analysis due to low GAPDH signal. **B)** Correlation between signal intensity of acetylated-α-tubulin, and docetaxel tumour accumulation in PC346C-DCC-K. Samples correspond to immunoblots depicted in Supplementary figure 4A, correlation was assessed by linear regression analysis. **C)** Immunoblots corresponding to Figure 2A, in which the impact of testosterone supplementation on α-tubulin acetylation was examined. Shown are the biological replicates of short-term docetaxel treated PC346C-DCC-K tumours obtained from castrate and testosterone supplemented mice.

Supplementary figure 5:Docetaxel induced cell death is impaired by testosterone. Representative confocal images of TUNEL staining in short-term docetaxel treated tumours obtained from castrate or testosterone supplemented mice (DocCx and DocTest resp.). Blue nuclear staining is Hoechst and green is TUNEL, scale bars represent 20 µm. The lower panels show an enlarged view.

Supplementary figure 6: AR stimulation does not affect docetaxel response in an AR negative prostate cancer cell line. Impact of androgen supplementation (0.1 nM R1881) on docetaxel response as compared to androgen deprived culture conditions (DCC) in the AR-negative PCa cell line PC339C (n=3). Docetaxel response was normalized to cell density at start of docetaxel treatment (dashed line) and plotted as relative cell expansion. Data was fitted using a non-linear curve fit, represented by the solid line.

Supplementary figure 7: Testosterone mediated AR-pathway activation overcomes docetaxel induced long-term tumour regression *in vivo*. A) Representative immunohistochemical stainings of the AR in tumours obtained after testosterone re-introduction, compared to docetaxel treated tumours in castrate mice (left panels). Scale bars represent 20 µm and 10 µm for the enlarged images (lower panels). Images were scored by two independent viewers for AR **(B)** percentage positive cells which was multiplied by the AR staining intensity (scale 0-3). **C)** Gene expression of the *AR* and *AR*-target genes expressed in PC346C-DCC-K dormant tumours after docetaxel treatment in castrate (n=4) and after testosterone re-introduction (both n=6). Heatmap depicts gene expression as assessed by qRT-PCR of the individual genes, with each row being one tumour sample. Plotted is the geomean of three technical replicates, normalized to two household genes and gene expression in tumours from castrate mice, using the $2^{-\Delta\Delta Ct}$ method.

Material and Methods

Cell culture

The human AR-positive CRPC cell line PC346C-DCC-K was derived from the human prostate cancer cell line PC346C, through long-term propagation in prostate growth medium (PGM) with 2% steroid stripped fetal calf serum (FCS) and in the absence of R1881[1,2]. The human AR-positive CRPC cell line VCaP-DCC-E was derived from the VCaP cell line through long-term propagation in RPMI with 10% steroid stripped FCS. DU145 docetaxel resistant cells (DU145-DR) were kindly provided by Zoran Culig and maintained as previously described[3]. Cell lines were characterized for the expression of prostate (cancer) transcripts (Supplementary figure 1), in short total RNA was isolated using RNA-Bee (Tel-Test, Friendwood, Texas, USA) according to the manufacturers protocol. qRT-PCR was performed as described previously[2], and gene-expression of the following targets was assessed; *AR*, *AR-V7*, *KLK3* (PSA) (using custom assays[2]), *SLCO1B3, ABCB1*, *TMPRSS2* and *FKBP5* and normalized to household genes *GAPDH* and *HMBS* (ThermoFisher Scientific, Hs00251986, Hs00184500, Hs01120965, Hs01561006, Hs99999905 and Hs00609296 resp.). Gene expression was normalized using the $2^{-\Delta Ct}$ method, DU145-DR was used as a positive control for ABCB1 expression. Mycoplasma testing was performed every 6 months, cell line authentication was performed by short tandem repeat analysis by the Promega PowerPlex 16 kit. PC346C-DCC-K cells were kept into culture for a maximum of 25 passages after initiating the castrate resistance phenotype, while the VCaP-DCC-E clone was kept in culture for 50 passages.

Docetaxel uptake in *SLCO1B3* expressing cells

For Docetaxel uptake assays we used Hek293T to over-express *SLCO1B3*. Hek293T cells were maintained in DMEM with 5% FCS. For drug uptake experiments, $1*10^5$ cells were plated in a 24-wells plate in phenol-red free medium (DMEM, ref # 31053-028, ThermoFisher Scientific, Waltham, Massachusetts, USA). After overnight incubation, cells were transfected with 0.5 µg of *SLCO1B3* plasmid DNA (GenScript, New Jersey, USA) or control vector (pCMV6-AC-IRES-GFP) using Lipofectamin 3000 (1 µl/µg DNA, ref # L3000015, ThermoFisher Scientific). Transfection efficiency was monitored visually by GFP expression, and uptake experiments were performed 24-48h after transfection. For competition assays, cells were incubated with phenol-red/FCS free media containing 200 µM testosterone or vehicle control for 15 minutes. Media was removed and cells were incubated with C^{14}-Docetaxel (1 µM, 56.27 µCi/mM) or ^{3}H-estradiol-17β-D-glucuronide (EbG; 0.2 µM) with or without testosterone for 10 minutes. After drug uptake, cells were washed three times with ice-cold PBS and lysed using 1 M NaOH at 4°C overnight. Cell lysates were neutralized using 2 M HCl and radioactivity measured using a scintillation counter (LS 6500 Counter, Beckman Coulter, Brea, California, USA). Radioactivity was corrected for protein concentration and activity of the drug solutions used. Uptake levels were normalized to vector control, or uptake in the absence of testosterone (vehicle control).

Animal Welfare

All animal experiments were approved by the Animal experiment committee under the Dutch experiments on Animal Act, with the reference number AVD101002017867. The current study is in compliance with the Arrive guidelines. Group size and experimental set-up was based on a previous study[2]. All operations (tumour inoculation, castration, blood sampling) are conducted under adequate anaesthesia to minimize animal discomfort. Subcutaneously growing tumours only cause mild discomfort.

Docetaxel activity *in vivo*

Forty, six weeks old, NMRI nu/nu male mice (Janvier, Le Genest Saint Isle, France) were subcutaneously inoculated with $5*10^6$ PC346C-DCC-K cells, while being anesthetized with isoflurane/O2. Tumour growth was observed after 2-4 weeks in ~95% of the mice. Once tumours reached a volume of 150 mm^3, mice were surgically castrated. Anaesthesia was provided by with Ketamine/Medetomidine (75 mg/kg and 1 mg/kg) and analgesia by Carprofen (5 mg/kg). After one week, mice were split into in two groups (based on tumour volume), to receive either a silastic implant with 40 mg of testosterone (ref #A0671, AppliChem, Darmstadt, Germany) or an empty implant to serve as control. During implantation of the silastic pellets, mice received anaesthesia by isoflurane/O2. The following day, mice were given an intravenous administration of docetaxel (33 mg/kg; 10% ETOH, 10% Tween-80 and 80% glucose solution, Sanofi, Paris, France) or NaCl as treatment control (inclusion was based on tumour volume). Overall we had four treatment groups with six mice each, docetaxel treatment or placebo control in castrate or testosterone supplemented mice. The experiment was repeated in 16 castrate mice, comparing docetaxel efficacy to placebo controls. Tumour volume was measured twice weekly by callipers and mice were followed until tumours exceeded a volume of 1500 mm^3, or a maximum follow up of 60 days after docetaxel treatment. At tumour volume 1500 mm^3 no tumour-related discomfort is observed, mice were euthanized by cervical dislocation. Other reasons for euthanizing the mice included continued weight loss, >15% loss in bodyweight in two days or >20% compared to start, and abnormal behaviour. Mice were maintained in an individually ventilated cage at 2-4 mice per cage, on a 12h dark/light cycle and cage enrichment was provided. Treatments or surgical procedures were initiated in the morning and food and water were provided ad libitum. Blood was sampled for prostate specific antigen (PSA) and testosterone analysis at tumour take, one week after testosterone (or empty control) supplementation and at end of the study. Blood plasma was isolated by centrifugation at 6800 RCF for 10 min and PSA was analysed by an electrochemiluminescence immunoassay (Cobas 8000, Roche, Basel, Switzerland). Plasma testosterone levels were analysed using a ChemiLuminescent Enzyme Immunoassay (Lumipulse G1200, Fujirebio, Gent, Belgium).

Docetaxel accumulation *in vivo*

Sixteen NMRI nu/nu male mice were inoculated subcutaneously with $5*10^6$ PC346C-DCC-K cells. When tumours reached a volume of 150 mm^3, mice were surgically castrated and after one week supplemented with testosterone-containing or empty silastic implant (based on tumour volume). Three days after intravenous administration of docetaxel (33 mg/kg), mice were sacrificed to determine docetaxel tumour accumulation. At least 40 mg of tumour sample was snap-frozen and used to determine docetaxel accumulation, which has been described previously[4,5].

Immunohistochemistry

Tumour tissue was formalin fixed paraffin embedded (FFPE) and 4 µm sections were stained for presence of the AR (1:200, SP107, Cell Marque, Rocklin, California, USA) and the cell cycle marker Ki67 (1:100, MIB-1, Agilent, Santa Clara, USA) using validated antibodies[6,7]. The secondary antibodies used were Goat anti-Rabbit and Goat anti-Rabbit/Mouse for the AR and Ki67 resp. (#P0448 and #K5007, Envision Dako Santa Clara, USA). Expression was visualized with DAB/H_2O_2 (EnVision kit, #K5007 Dako) and sections were counterstained with haematoxylin. For all tissue sections stained we included one sample with the secondary antibody (goat anti-Rabbit/Mouse) only, to visualize potential a-specific staining. Images were obtained using the Olympus BX41 microscope equipped with 2x, 10x, 20x and 40x UPlanFL N objectives, a ColorView III camera and CellB imaging software (version 3.4, Olympus, Shinjuku, Tokyo, Japan). All IHC stainings were blinded for treatment and quantified by two independent persons based on two or three representative images. Ki67 was scored based on percentage positive tumour cells only while AR score was calculated by multiplying percentage positive tumour cells with the staining intensity score (0-3).

TUNEL staining

Cell-death was assessed using TdT-mediated dUTP-X nick end labelling (TUNEL). Four µm FFPE sections were stained according to manufacturer's protocol (ref #11684795910, Merck, Darmstadt, Germany), and counterstained with Hoechst (ref # H3570, ThermoFisher Scientific). Tumour sections were blinded to treatment group and imaged using a Leica fluorescent microscope (DM4000b, Nussloch, Germany). Ten fields per tumour sample were imaged at a 400x magnification and quantification was performed as described previously [8]. Representative images were obtained using the SP5 Leica confocal microscope at a 630x magnification (HCX PL APO CS objective with Photo Multiplier Tube detector and LAS X imaging software).

AR-pathway expression upon testosterone stimulation *in vivo*

Tumour tissue was lysed and homogenized in QIAzol (ref #79306, Qiagen, Hilden, Germany) using an Ultra-Turrax T25 (Janke & Kunkel, Staufen, Germany). Total RNA was isolated using the miRNA-easy mini kit (ref # 217004, Qiagen), and RNA quality was measured using the Bioanalyzer RNA 6000 Nano assay (ref #5067, Agilent, Santa Clara, California, USA). All RNA samples had a RIN value ≥7. qRT-PCR was performed as described

previously, gene-expression was normalized to household genes (*GAPDH* and *HMBS*) and gene expression in castrate tumours using the $2^{-\Delta\Delta Ct}$ method.

Tubulin stabilization by docetaxel treatment *in vivo*

Tumours tissue was lysed and homogenized in RIPA buffer (0.01 M Tris-HCl, 0.05 M NaCl, 0.05% deoxycholate, 0.1% sodiumdodecyl sulfate (SDS), 5 mM ethylenediaminetetraacetic acid (EDTA) and 1% Triton x-100) supplemented with protease and phosphatase inhibitors (ref #78429, Halt™ Protease Inhibitor Cocktail and ref# 78420 Halt™ Phosphatase Inhibitor Cocktail both Thermo Fisher Scientific), using an Ultra-Turrax T25. Protein homogenates were separated from the nucleic acids by centrifugation (4°C, 15 min at 20.000 g). Protein concentration was measured by the Pierce BCA protein assay (ref #23227, ThermoFisher Scientific), and 10 µg of protein lysate was used for immuno-blotting. Tubulin stabilization was visualized by staining for acetylated-α-tubulin (1:1000, ref #6-11B-1, Santa Cruz, Dallas, USA) and imaged using chemiluminescence (ref #11500694001, Merck). Staining intensity was assessed by the Odyssey Li-COR (C-Digit model 3600, Lincoln, Nebraska, USA) imaging system and normalized to GAPDH (1:10.000, ref #SC-47724, Santa Cruz) signal intensity.

Cell viability assay

For cell viability assays PC346C-DCC-K and VCaP-DCC-E cells were plated at a cell density of $5*10^3$ cells per well in a 96-wells plate. VCaP-DCC-E cells were maintained in RMPI containing 10% DCC serum, while PC346C-DCC-K cells were maintained in PGM-basic medium [9]. After overnight incubation, cells were exposed to a dose-range of docetaxel (0-100 nM, Sanofi) with or without the addition of 0.1 nM R1881, using six replicates per condition. After 10 days cell viability was measured by MTT assay, as described previously [9], and normalized to values of six untreated wells at day one. The docetaxel sensitivity of the non-adherent, *AR* and *SLCO1B3* negative cell line PC339C[10] was examined using the PrestoBlue assay (ref #A13261, ThermoFisher Scientific). The experimental set-up was similar to the MTT assay described above, with the following alteration, cells were plated at a cell density of $2.5*10^3$ and docetaxel response was measured after 7 days.

Statistical analysis

Kaplan-Meier plots and log-rank survival analysis as depicted in figure 1A, were performed using the survival package version 3.1-6 in Rstudio Version 1.1.463[11,12]. A pairwise comparison with Bonferroni correction was used to compare survival in the independent groups. The boxplot graphs shown throughout the manuscript were produced using ggplot2 version 3.2.1 in Rstudio[13], the hinges of the boxplots represent the 25th and 75th percentile with the median and the whiskers represent 1.5x the interquartile range, individual data points were plotted on the boxplots using ggbeeswarm[14]. Statistical analysis of uptake and subsequent competition assays was performed using an one-way Anova with Dunn's post-test for multiple comparisons using Rstudio[15]. The frequency of TUNEL positive cells after treatment (Figure 2B) was analysed using an one-way Anova with Tukey post-test for multiple comparisons using Rstudio. The relation between acetylated-α-tubulin staining intensity as obtained by immunoblotting and docetaxel tumour accumulation was analysed using linear regression

analysis in GraphPad Prism (version 5.01, GraphPad Software, San Diego, California, USA). The cell viability data was analysed using non-linear fit in GraphPad, we used a log(inhibitor) versus response and variable slope to compare EC50 and maximum effect in castrate and androgen supplemented conditions. The impact of testosterone supplementation on plasma PSA, testosterone levels, docetaxel tumour accumulation and AR nuclear localization or Ki67 expression was assessed using a two-sided T-test in Rstudio.

References

1 Marques, R. B., van Weerden, W. M., Erkens-Schulze, S., de Ridder, C. M., Bangma, C. H., Trapman, J. *et al.* The human PC346 xenograft and cell line panel: a model system for prostate cancer progression. *Eur Urol* **49**, 245-257 (2006).

2 Mout, L., de Wit, R., Stuurman, D., Verhoef, E., Mathijssen, R., de Ridder, C. *et al.* Testosterone Diminishes Cabazitaxel Efficacy and Intratumoral Accumulation in a Prostate Cancer Xenograft Model. *EBioMedicine* **27**, 182-186 (2018).

3 O'Neill, A. J., Prencipe, M., Dowling, C., Fan, Y., Mulrane, L., Gallagher, W. M. *et al.* Characterisation and manipulation of docetaxel resistant prostate cancer cell lines. *Mol Cancer* **10**, 126 (2011).

4 de Morree, E., van Soest, R., Aghai, A., de Ridder, C., de Bruijn, P., Ghobadi Moghaddam-Helmantel, I. *et al.* Understanding taxanes in prostate cancer; importance of intratumoral drug accumulation. *Prostate* **76**, 927-936 (2016).

5 Engels, F. K., Mathot, R. A., Loos, W. J., van Schaik, R. H. & Verweij, J. Influence of high-dose ketoconazole on the pharmacokinetics of docetaxel. *Cancer Biol Ther* **5**, 833-839 (2006).

6 Varun, K., Jianjun, Y., Vernon, P., Iulia Cristina, T., Amy, P. & Hirdesh, U. Androgen Receptor Immunohistochemistry as a Companion Diagnostic Approach to Predict Clinical Response to Enzalutamide in Triple-Negative Breast Cancer. *JCO Precision Oncology* 10.1200/po.17.00075, 1-19 (2017).

7 Lindboe, C. F. & Torp, S. H. Comparison of Ki-67 equivalent antibodies. *J Clin Pathol* **55**, 467-471 (2002).

8 Zhang, W., van Weerden, W. M., de Ridder, C. M. A., Erkens-Schulze, S., Schonfeld, E., Meijer, T. G. *et al.* Ex vivo treatment of prostate tumor tissue recapitulates in vivo therapy response. *Prostate* **79**, 390-402 (2019).

9 Marques, R. B., Erkens-Schulze, S., de Ridder, C. M., Hermans, K. G., Waltering, K., Visakorpi, T. *et al.* Androgen receptor modifications in prostate cancer cells upon long-termandrogen ablation and antiandrogen treatment. *Int J Cancer* **117**, 221-229 (2005).

10 de Morree, E. S., Bottcher, R., van Soest, R. J., Aghai, A., de Ridder, C. M., Gibson, A. A. *et al.* Loss of SLCO1B3 drives taxane resistance in prostate cancer. *Br J Cancer* **115**, 674-681 (2016).

11 R: A Language and Environment for Statistical Computing v. Version 1.1.463 (R Foundation for Statistical Computing, Vienna, Austria, 2019).

12 A Package for Survival Analysis in S v. version 2.38 (2015).

13 Ginestet, C. ggplot2: Elegant Graphics for Data Analysis. *J R Stat Soc a Stat* **174**, 245-245 (2011).

14 ggbeeswarm: Categorical Scatter (Violin Point) Plots v. R package version 0.6.0.

15 dunn.test: Dunn's Test of Multiple Comparisons Using Rank Sums. v. R package version 1.3.5. (2017).
